# Ongoing multi-country outbreak of carbapenem-resistant *Enterobacter hormaechei* ST1344 carrying *bla*_NDM-5_ in the European Union/European Economic Area, March 2025 to April 2026

**DOI:** 10.2807/1560-7917.ES.2026.31.25.2600460

**Published:** 2026-06-25

**Authors:** Louise Roer, Mirco Sandfort, Sebastian Haller, Sarah A Roth, Lisa-Marie Höfken, Soheila Javadi, Núria Balanza, Amelie Friedsam, Silver A Wolf, Claus Østergaard, Monica T Y Wong, Joanna Lis-Tønder, Annelot F Schoffelen, Martin Cormican, Lone Jannok Porsbo, Christina Clarke, Henrik Hasman, Heidrun Kerschner, Anette M Hammerum, Rainer Hart, Ørjan Samuelsen, Miriam Sare, Marie Meo, Anne-Sophie Lagneaux, Monique Perrin, Vilhelm Müller, Sandra Witteveen, Sabine C de Greeff, Daan W Notermans, Olov Svartström, Erik Alm, Marius Linkevicius, Anke Kohlenberg, Antoni P A Hendrickx

**Affiliations:** 1 National Reference Laboratory for Antimicrobial Resistance, Department of Bacteria, Parasites and Fungi, Statens Serum Institut, Copenhagen, Denmark; 2 European Union Reference Laboratory for Public Health on Antimicrobial Resistance in Bacteria (EURL-PH-AMR), Copenhagen, Denmark; 3 Department of Infectious Disease Epidemiology, Robert Koch Institute, Berlin, Germany; 4 Public Health Agency of Lower Saxony (NLGA), Hanover, Germany; 5 German National Reference Centre for Multidrug-resistant Gram-negative Bacteria, Department of Medical Microbiology, Ruhr-University Bochum, Bochum, Germany; 6 Centre for Infectious Disease Control, National Institute for Public Health and the Environment (RIVM), Bilthoven, the Netherlands; 7 ECDC Fellowship Programme, Field Epidemiology path (EPIET), European Centre for Disease Prevention and Control (ECDC), Stockholm, Sweden; 8 Postgraduate Training for Applied Epidemiology (PAE), Department for Infectious Disease Epidemiology, Robert Koch Institute, Berlin, Germany; 9 Genome Competence Centre (MF 1), Robert Koch Institute, Berlin, Germany; 10 Department of Clinical Microbiology, Lillebaelt Hospital, Vejle, Denmark; 11 School of Medicine, University of Galway, Galway, Ireland; 12 National Center for Infection Control, Infectious Disease Epidemiology & Prevention, Statens Serum Institut, Copenhagen, Denmark; 13 Galway Reference Laboratory Service, Galway, Ireland; 14 National Reference Center for Antimicrobial Resistance, Linz, Austria; 15 Norwegian Centre for Detection of Antimicrobial Resistance, Department of Microbiology and Infection Control, University Hospital of North Norway, Tromsø, Norway; 16 Department of Infection Control and Preparedness, Norwegian Institute of Public Health, Oslo, Norway; 17 Laboratoire National de Santé, Dudelange, Luxembourg; 18 Public Health Agency of Sweden, Solna, Sweden; 19 European Centre for Disease Prevention and Control (ECDC), Stockholm, Sweden; * These authors contributed equally to this work and share first authorship.

**Keywords:** “carbapenem-resistant Enterobacterales”, “carbapenemase”, “surveillance”, “disease outbreaks”, “cross-border spread”, “drug resistance, bacterial”, New Delhi metallo-β-lactamase

## Abstract

In seven EU/EEA countries, the emergence of NDM-5-producing *Enterobacter hormaechei* ST1344 among patients has been detected. In minimum spanning tree analysis of core genome-multilocus sequence-typing (cgMLST) profiles, isolates cluster with ≤ 4 allelic differences. The outbreak includes 57 cases (29 female/28 male; median age: 73 years), which occurred between March 2025 and April 2026. Many were hospitalised (n = 45), some with extensive prior medical treatment, and only three reported travel abroad. A cross-border investigation, including coordinated exposure questionnaires, has not yet identified a source.

On 12 September 2025, the National Institute for Public Health and the Environment in the Netherlands posted an alert in EpiPulse, the European surveillance portal for infectious diseases, (https://www.ecdc.europa.eu/en/publications-data/epipulse-european-surveillance-portal-infectious-diseases) on a sudden emergence of infections/colonisations with *Enterobacter hormaechei* sequence type (ST) 1344 carrying *bla*_NDM-5_ on an IncX3-plasmid, with no epidemiological links between patients. This prompted a search for related cases in European Union (EU)/European Economic Area (EEA) countries. Up to May 2026, 57 cases were confirmed by core genome multilocus sequence typing (cgMLST) [1,2] as part of an outbreak affecting seven countries with the earliest case occurring in March 2025. Here, we describe the cross-border emergence of the outbreak strain to motivate and support the identification of its source, as well as to raise awareness of its spread and of the importance of continued case detection.

## Confirmation of a cross-border outbreak

**Figure 1:**
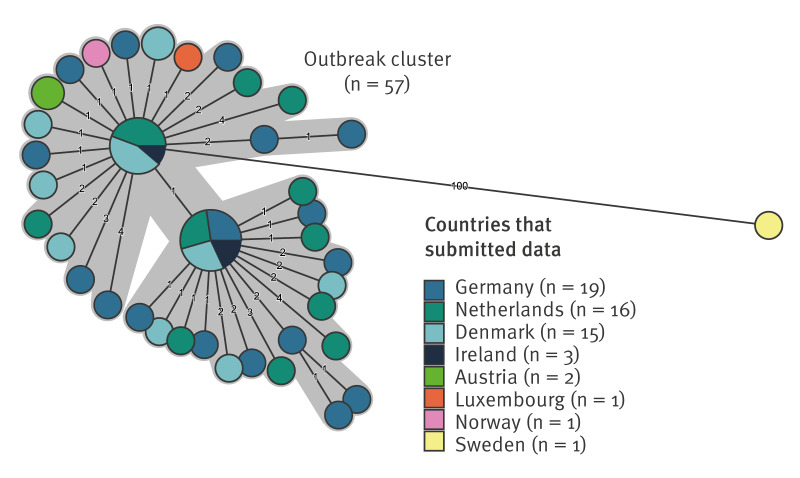
Minimum spanning cgMLST-based tree with *Enterobacter hormaechei* ST1344 collected in eight EU/EEA countries, March 2025–April 2026 (n = 58 isolates)

Following the alert, countries participating in the EU/EEA Antimicrobial Resistance Genes Surveillance Network (EURGen-Net) were requested to report similar isolates and share genomic and epidemiological data. Up to April 2026, 17 countries responded, with eight reporting *E. hormaechei* ST1344, as shown in Supplementary Table 1. All isolates carried *bla*_NDM-5_ except one from Sweden. For 15 of the 22 isolates with reported MIC data (Etest), the minimum inhibitory concentration (MIC) for meropenem was ≥ 32 mg/L indicating high-level carbapenem-resistance; Supplementary Table 2 further describes antimicrobial susceptibility testing (AST) profiles for the Dutch isolates. The European Centre for Disease Prevention and Control (ECDC) and the EU Reference Laboratory for Public Health on Antimicrobial Resistance in Bacteria (EURL-PH-AMR) performed species-specific cgMLST analyses [1,2]. This revealed a genetic cluster of highly related isolates from seven countries (Figure 1), confirming a multi-country outbreak and leading to a joint investigation. Suspected outbreak cases were defined as individuals infected/colonised with *E. hormaechei* ST1344. Cases were confirmed if their respective sequenced isolates carried the New Delhi metallo-β-lactamase (NDM)-5-gene and clustered with 10 or less allelic differences to their closest outbreak-related isolate in the cgMLST cluster analysis.

cgMLST: core-genome multilocus sequence typing; EU/EEA: European Union/European Economic Area; MST: minimum spanning tree; ST: sequence type.

The nodes in the MST represent *E. hormaechei* isolates from different countries, which are depicted in different colours. Large(r) nodes represent ˃ 1 isolates without cgMLST allelic difference. The length of lines between the nodes reflects the number of allelic differences between isolates in the respective nodes. These differences are also quantified with numbers figuring on top of the lines. Isolates with ≤ 10 allelic differences between them form a cluster.

## Genomic analysis

The 58 suspected case isolates of *E. hormaechei* ST1344 reported by eight countries were included in the genomic analysis. Confirmed case isolates with cgMSLT cluster type (CT) 7 and *bla*_NDM-5_ from 57 cases were included as part of the outbreak cluster from Germany (n = 19), the Netherlands (n = 16), Denmark (n = 15), Ireland (n = 3), Austria (n = 2), Luxembourg (n = 1), and Norway (n = 1) between March 2025 and April 2026. The 57 outbreak isolates formed a single-linkage cluster with 0–4 cgMLST allelic differences between nearest neighbours. A single nt-polymorphism (SNP)-based comparison supported the high genetic relatedness of the confirmed isolates (0–9 pairwise SNP) and showed no patterns by country, specimen type, or sampling time, as depicted in Supplementary Figure 1 and described in Supplementary Table 3. Retrospective screening of carbapenemase-producing *Enterobacter cloacae*-complex isolates at the national reference laboratories from Norway since 2007, Denmark since 2014, the Netherlands since 2012, Ireland since 2017, or from Germany since 2024 identified no earlier occurrence of *bla*_NDM-5_-carrying *E. hormaechei* ST1344. 

The non-*bla*_NDM-5_ carrying isolate from Sweden and 16 publicly available *E. hormaechei* ST1344 genomes from the National Center for Biotechnology Information (NCBI) Pathogen Detection database (accessed on 12 May 2026) were included for comparison. Seven genomes from the outbreak period, one from the United Kingdom (UK) and six from the United States (US), clustered with the outbreak isolates by cgMLST as illustrated in Supplementary Figure 2. Collection of epidemiological metadata for these isolates is ongoing, and they have not been included in the epidemiological analysis and outbreak case count.

Hybrid assemblies of representative Dutch and Danish isolates identified *bla*_NDM-5_ on a 45.3-kb IncX3-plasmid, a type associated with horizontal transfer [3]. Additional resistance genes were located on a larger 281-kb IncHI2/IncHI2A-plasmid, including *aac(3)-IIa*,* aac(6')-Ib-cr*,* aadA1*,* aph(3”)-Ib*,* aph(6)-Id*,* bla*_CTX-M-15_, *bla*_OXA-1_, *bla*_TEM-1B_, *catA1*,* catB3*,* dfrA14*,* qnrB1,* and *sul2.* Chromosomal resistance determinants included *bla*_ACT-16_, encoding an AmpC-type β-lactamase, and *fosA*. A plasmid-specific marker was subsequently used to screen short-read data, as described in Supplementary methods, and identified the outbreak-associated IncX3-plasmid in confirmed case *E. hormaechei* isolates and in additional Enterobacterales species. In Denmark, the plasmid marker was detected in additional Enterobacterales species sampled from four patients between May and October 2025, including three confirmed outbreak cases and one patient without detection of *E. hormaechei*. Additional plasmid-positive species included *Escherichia coli*, *Serratia sarumanii*, *Klebsiella aerogenes*, *K. pneumoniae,* and *K. quasipneumoniae*. In the Netherlands, the plasmid was detected in July 2025 in *K. pneumoniae* from a confirmed case, and in two patients without detection of *E. hormaechei* in July and October 2025 involving *K. pneumoniae* and *E*. *coli*, respectively. No information on additional plasmid-positive species was provided by the other countries with outbreak cases.

## Epidemiological investigation

**Figure 2:**
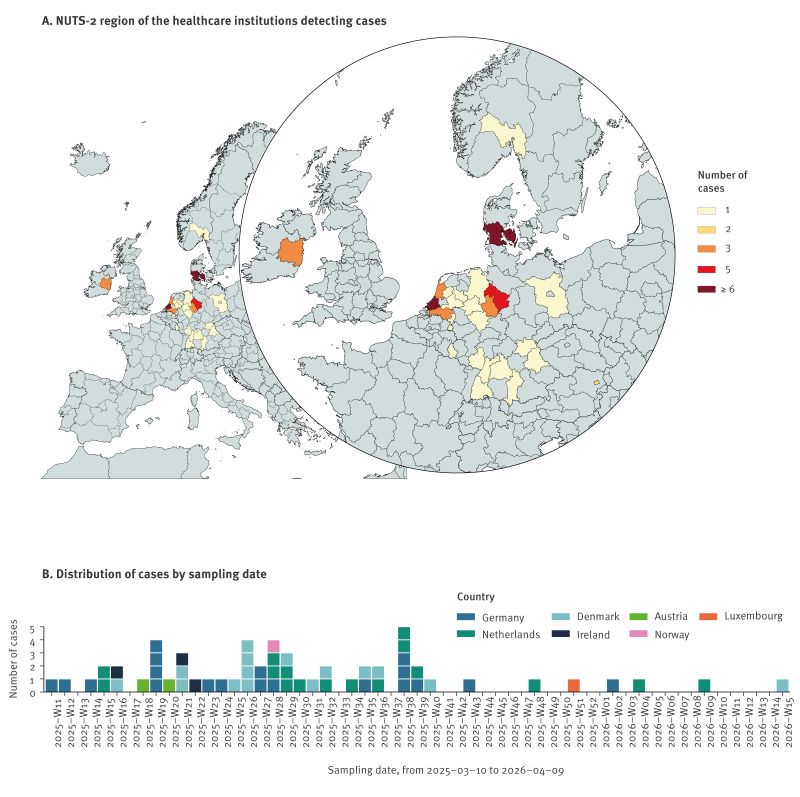
(A) Map indicating the regions of healthcare institutions with confirmed cases and (B) graph showing the distribution of confirmed cases by sampling date of *Enterobacter hormaechei* ST1344-CT7 carrying *bla*_NDM-5_ cases in seven EU/EEA countries, March 2025–April 2026 (n = 57 confirmed cases)

CT: cluster type; EU/EEA: European Union/European Economic Area; NUTS-2: Nomenclature of Territorial Units for Statistics; ST: sequence type.

Case information was compiled from national surveillance data, collected via a harmonised template, as shown in Supplementary Table 4. Case location was defined as the Nomenclature of Territorial Units for Statistics (NUTS)-2 region of the healthcare institution detecting the case or, if missing, the cases’ residence. Cases occurred in 26 NUTS-2 regions (Figure 2A), with highest case counts in Southern Denmark (all 15 cases from Denmark), followed by one region in the Netherlands and one in Germany. The earliest sample date was in March 2025 for two cases from Germany (Figure 2B) and samples corresponding to cases continued to occur until April 2026.

**Figure 3:**
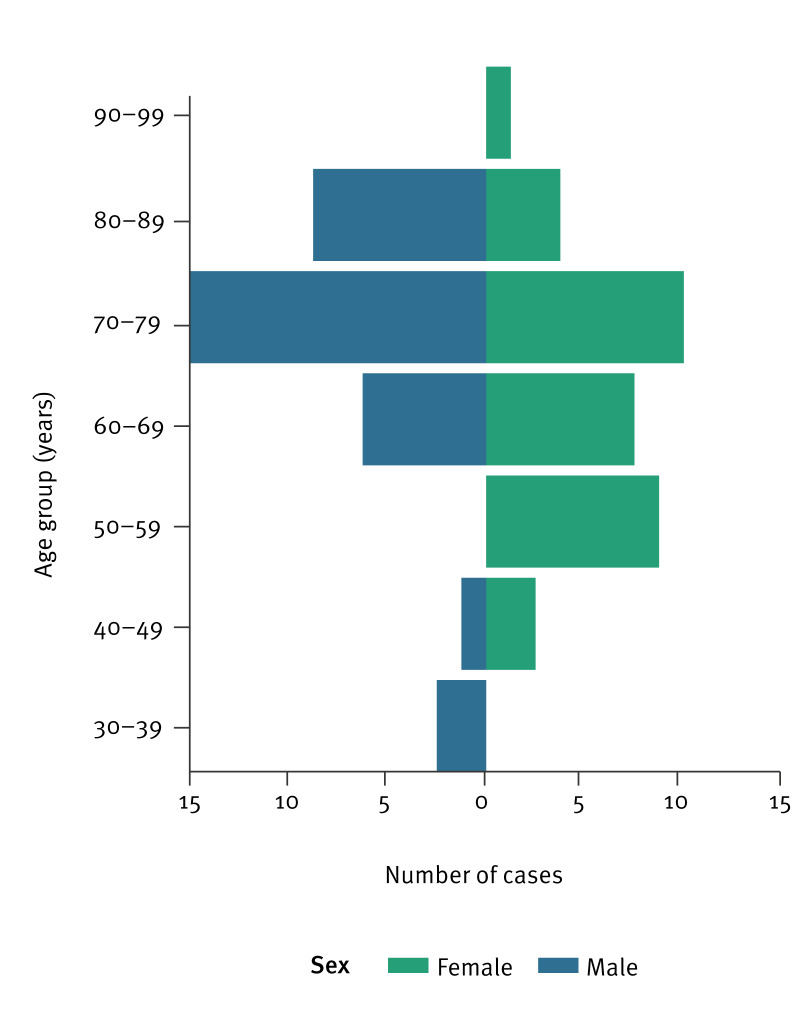
Distribution by age and sex of *Enterobacter hormaechei* ST1344-CT7 carrying *bla*_NDM-5_ cases in seven EU/EEA countries, March 2025–April 2026 (n = 57)

CT: cluster type; EU/EEA: European Union/European Economic Area; ST: sequence type.

There were 29 female and 28 male cases (Table, Figure 3) and, overall, cases had a median age of 73 years, spanning 37–94 years. Travel to another country within 12 months prior to the obtention of a sample with the outbreak strain was reported for only three cases. Of 52 cases with available information, 45 were inpatients at sampling, partially with extensive medical treatment histories. Among the 45 inpatient cases, acquisition for 19 was labelled as presumably healthcare-associated (typically when first positive sampling occurred 48 h after admission) based on the countries’ routine surveillance data. Seven of the 52 overall cases were outpatients, of which four had previous hospitalisation within 12 months explicitly excluded. The seven outpatient cases included three in Germany, two in the Netherlands, one in Denmark, and one in Luxembourg. Two of the outpatient cases in Germany and the ones in the Netherlands were detected in each of the two regions having the most cases within these respective countries. Among 57 cases with available data, sampling was conducted via rectal swabs for 18 cases, while a urine sample was obtained for 21, followed by wound for six, blood for five, other clinical specimens for four and soft tissue for two. Further case characteristics, including timeline of healthcare visits and exposure to products (e.g. antibiotics, ultrasound gels, or non-alcoholic disinfectants), were collected via a joint questionnaire and are currently being analysed.

**Table:** Epidemiological characteristics of cases with *Enterobacter hormaechei* ST1344-CT7 carrying *bla*_NDM-5_ in seven EU/EEA countries, March 2025–April 2026 (n = 57)

Characteristic	Number of cases
Sex	
Female	29
Male	28
Missing	0
Hospitalisation	
Inpatient	45
Outpatient	7
Missing	5
Assumed acquisition among 45 inpatient cases	
Healthcare-associated	19
Missing or unknown	26
Sample material	
Urine	21
Wound	6
Blood	5
Soft tissue	2
Other clinical specimen	4
Rectal screening specimen	18
Missing	1
Travel within 12 months prior to the date when the sample with the outbreak strain was obtained	
Yes, travel to Germany	1
Yes, travel to Spain	1
Yes, travel to Greece	1
No	12
Missing or unknown	42

CT: cluster type; EU/EEA: European Union/European Economic Area; ST: sequence type.

Among the 45 inpatient cases, 36 had no concomitant stay in the same hospital ward as another case. The remaining nine cases from two Danish departments, located at two different hospitals within the same region, were partially confirmed as being part of a local healthcare-associated outbreak. Infection prevention and control measures were implemented in accordance with national carbapenemase-producing organisms’ guidelines, including isolation of carbapenemase-producing Enterobacterales (CPE)-positive patients, screening of contact patients, and enhanced cleaning of the affected ward. Excluding these cases did not alter the overall distribution of case characteristics.

## Discussion

We continue to investigate an outbreak of *E. hormaechei* ST1344-CT7 carrying *bla*_NDM-5_ in seven EU/EEA countries. The concomitant multi-country emergence and genetic stability suggest a persistent, inanimate source, potentially available across the EU/EEA, the UK and the US. Previously described sources in *E. cloacae*-complex outbreaks were mainly medicines/medicinal products such as dicloxacillin [4], sodium chloride solutions [5], or ultrasound gel [6]. Hence, collating exposure information focused on such products. Preliminary analyses have not yet pointed out a specific product, compound, or manufacturer beyond common medical exposures such as antibiotics, ultrasound gels, or non-alcoholic disinfectants.

It should be noted that the presented data likely represent an underestimation of the total number of cases, even if we already assumed additional cases from the NCBI genomes or from non*-E. hormaechei* Enterobacterales, which have the outbreak characteristic IncX3 plasmid carrying *bla*_NDM-5_ gene and originate from patients without a confirmed *E. hormaechei* isolate. Clinical species determination often stops at the *E. cloacae-*complex level, which is a commonly detected taxonomic group among CPE [7], resulting in no submission for central genotyping and in the potential outbreak association possibly being missed. Second, CPE detection is mainly limited to hospitals during risk-adapted on-admission screening, or intensive care unit transfer screening, or as part of clinical diagnostics [8]. Many cases being inpatients might thus be a detection bias. Most cases lacked typical risk factors for CPE screening such as recent travel. Furthermore, differentiating healthcare-associated vs community acquisition is generally challenging. Lastly, exposure to products is difficult to assess as this is not always documented at patient-level [9].

## Conclusion

We report an ongoing cross-border outbreak of NDM-5-producing *E. hormaechei* ST1344 with the most recent confirmed case detected in Denmark in early April. Patterns in case occurrence and characteristics would most plausibly be explained by a common, inanimate acquisition or infection source, possibly a medicine, medical product, or a product that does not require prescription, but no source has been identified to date. Data sharing is a prerequisite for such investigations, and EpiPulse has proven to be a valuable tool for communication on multi-country outbreaks. Systems for efficient traceability of medicines and medical products are needed. Since completion of the analyses presented here, an additional suspected outbreak case has been identified in Austria in May 2026. As the cause of the outbreak remains unknown, this event indicates that case occurrence may continue, and a persistent common acquisition source cannot be ruled out. Hence, alertness for new case detection among both inpatients and outpatients is warranted and carbapenem-resistant *E. cloacae-*complex isolates should be tested for NDM production and outbreak relatedness.

## Data Availability

The raw short-read and long-read sequence data generated and analysed in this study are available in the Sequence Read Archive (SRA) in the Bioprojects depicted in Supplementary Table 1. The authors confirm that the supporting data, protocols, and accession numbers have been provided.

## Supplementary Data

166000 Supplementary figures

## Supplementary Data

218000 Supplementary methods

## Supplementary Data

73000 Supplementary tables
