# Supplementary material for: Ongoing multi-country outbreak of carbapenem-resistant Enterobacter hormaechei ST1344 carrying blaNDM-5 in the European Union/European Economic Area, March 2025 to April 2026
Source: Euro Surveill. 2026 Jun 25;31(25):2600460. doi: 10.2807/1560-7917.ES.2026.31.25.2600460 (PMC13309760; doi:10.2807/1560-7917.ES.2026.31.25.2600460)
Supplement: Supplementary figures [file 26-00460_Supplementary_figures.pdf]

This supplementary material is hosted by *Eurosurveillance* as supporting information alongside the article Ongoing multi-country outbreak of carbapenem-resistant *Enterobacter hormaechei* ST1344 carrying *bla*NDM-5 in the European Union/ European Economic Area, March 2025 to April 2026, on behalf of the authors, who remain responsible for the accuracy and appropriateness of the content. The same standards for ethics, copyright, attributions and permissions as for the article apply. Supplements are not edited by *Eurosurveillance* and the journal is not responsible for the maintenance of any links or email addresses provided therein.

**SUPPLEMENTARY FIGURE 1.** Single nucleotide-polymorphism based genomic comparison of the *Enterobacter hormaechei* ST1344 cluster across seven EU/EEA countries, March 2025–April 2026 (n = 57 confirmed cases).
From left: maximum-likelihood phylogeny rooted using an outgroup (not shown), demonstrating close genetic relatedness among isolates (0–9 SNP differences), with each isolate differing by at most 6 SNPs from its nearest neighbour, based on mapping to the first isolate as reference.
Middle: coloured strips indicate country, sample type (clinical or screening), and clinical specimen source type, as defined in the legends. Right: sampling timeline by week, from the first isolate in week 11, 2025, to the most recent isolate in week 15, 2026.

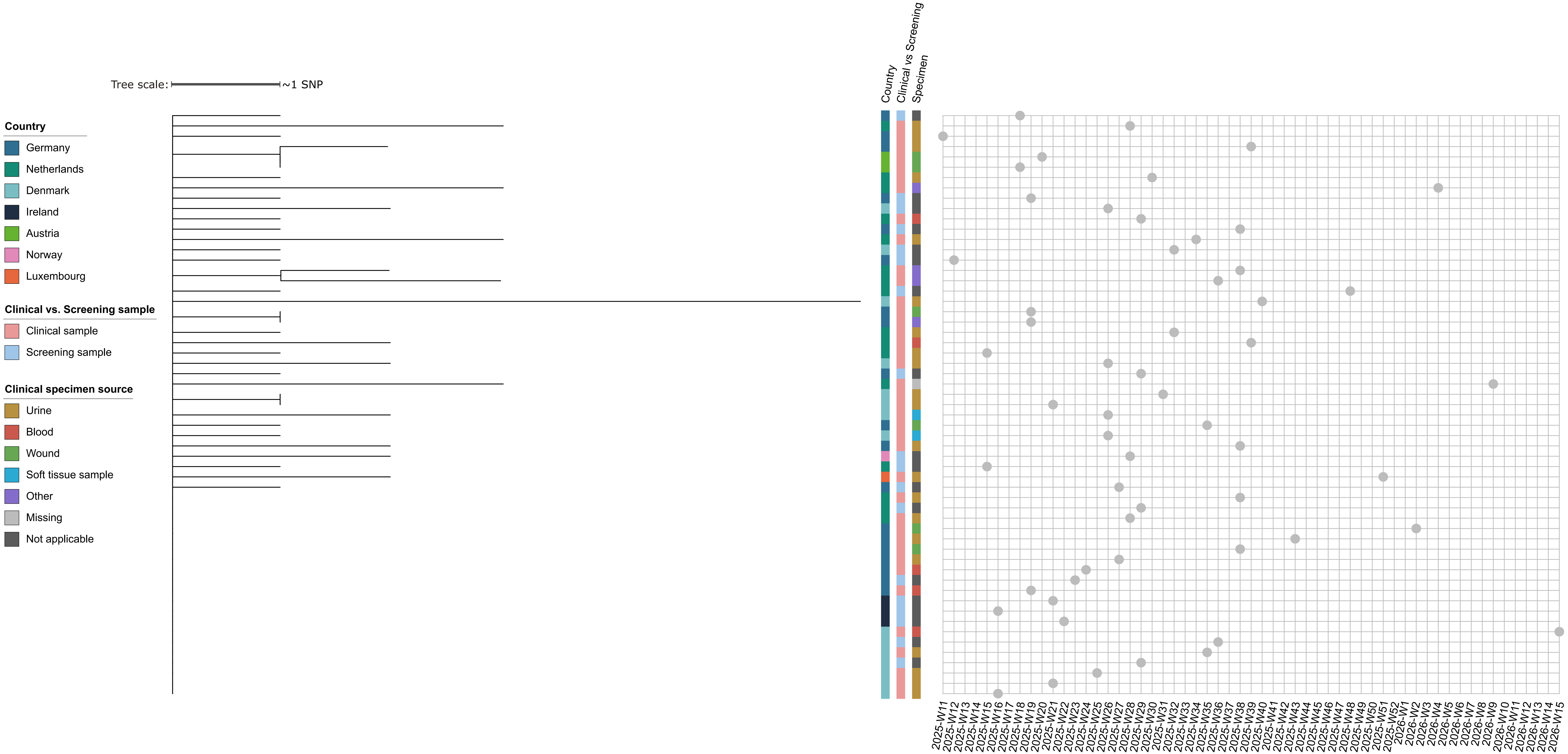

**SUPPLEMENTARY FIGURE 2.** Minimum spanning cgMLST-based tree with *Enterobacter hormaechei* ST1344 NCBI isolates (n = 16), as well as isolates collected in eight EU/EEA countries (n = 58), March 2025–April 2026 (n = 74 total isolates).

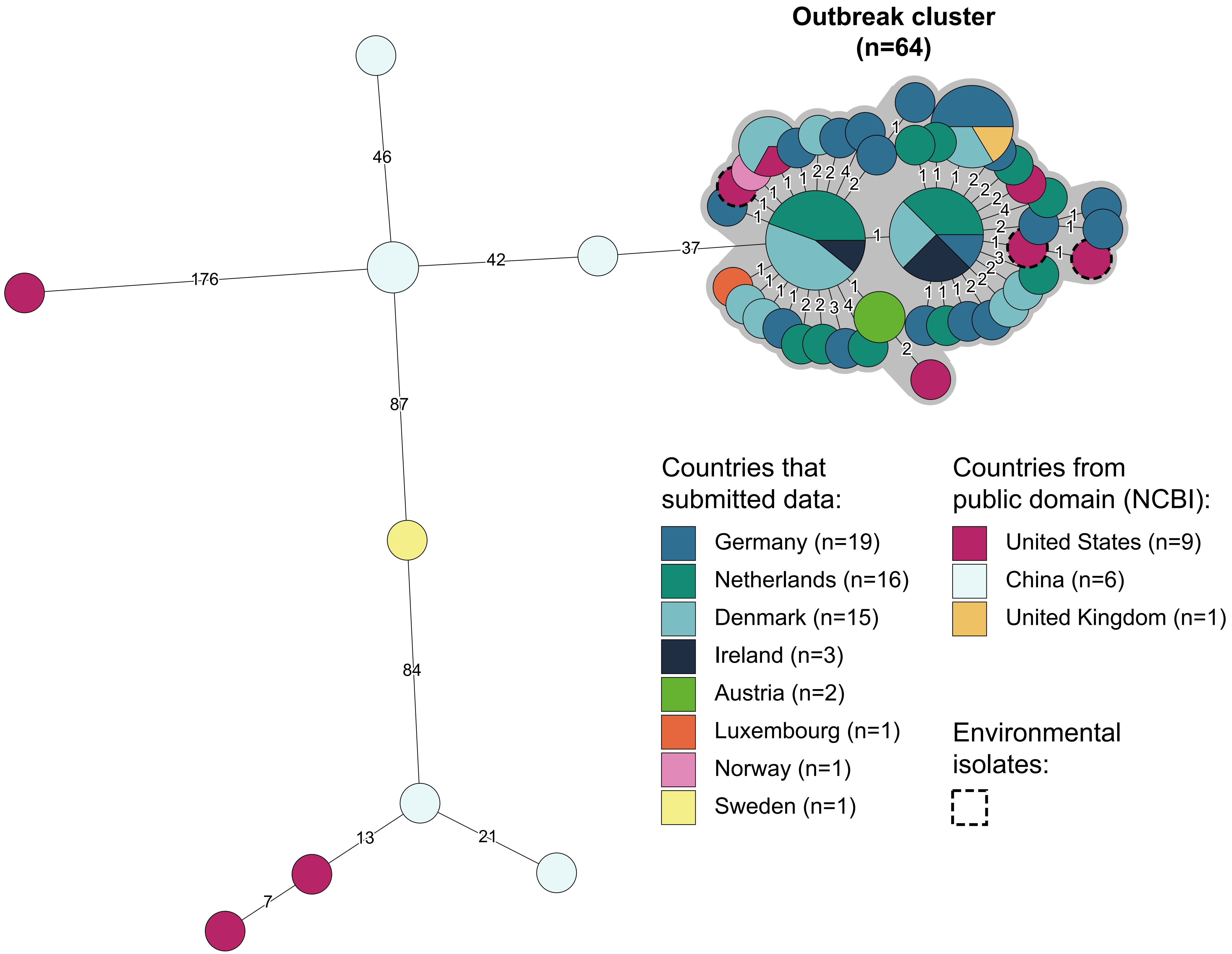


EU/EEA: European Union/European Economic Area; NCBI: National Center for Biotechnology Information

GenBank genome assembly accession numbers (NCBI) included in the phylogenetic analysis. Genomes from United States (n = 6) and the United Kingdom (n = 1) clustering with the study isolates: GCA_053080565.1, GCA_053108725.1, GCA_053156875.1, GCA_053156915.1, GCA_053909705.1, GCA_053909865.1, and GCA_981475375.1. Genomes included in the analysis but not clustering with the study isolates: GCA_005217155.2, GCA_021285985.1, GCA_023750265.1, GCA_023750525.1, GCA_023754115.1, GCA_036912015.1, GCA_049237945.1, GCA_050102415.1, and GCA_050910065.1.
