# Supplementary material for: Ongoing multi-country outbreak of carbapenem-resistant Enterobacter hormaechei ST1344 carrying blaNDM-5 in the European Union/European Economic Area, March 2025 to April 2026
Source: Euro Surveill. 2026 Jun 25;31(25):2600460. doi: 10.2807/1560-7917.ES.2026.31.25.2600460 (PMC13309760; doi:10.2807/1560-7917.ES.2026.31.25.2600460)
Supplement: Supplementary methods [file 26-00460_Supplementary_methods.pdf]

Supplementary methods

This supplementary material is hosted by *Eurosurveillance* as supporting information alongside the article Ongoing multi-country outbreak of carbapenem-resistant *Enterobacter hormaechei* ST1344 carrying *bla*$_{NDM-5}$ in the European Union/European Economic Area, March 2025 to April 2026, on behalf of the authors, who remain responsible for the accuracy and appropriateness of the content. The same standards for ethics, copyright, attributions and permissions as for the article apply. Supplements are not edited by *Eurosurveillance* and the journal is not responsible for the maintenance of any links or email addresses provided therein.

Whole genome sequencing assembly

Short-read sequences were assembled using SPAdes v3.15.5 (1). Long-read sequencing reads were used in hybrid assemblies with short-reads using different pipelines per country.

Typing and classification

MLST profiles were determined with MLSTFinder (2), based on the pubMLST database (3). Resistance genes and plasmid replicons were identified using ResFinder v.4.7.2 (database v.2.6.0) (4) and PlasmidFinder v2.0.1 (database from 1 March 2023) (5).

cgMLST and SNP analysis

Alleles were called using ChewBBACA v3.3.10 (6). Minimum spanning tree was constructed using the Ridom cgMLST scheme (7,8) and visualized using BioNumerics 7.6.3 (Applied Maths NV/bioMérieux). Single nucleotide-polymorphism (SNP) analyses were performed using the Northern Arizona SNP Pipeline v. 1.2 (NASP) (9), with the earliest outbreak isolate from Germany as reference. Phylogenetic analyses were performed by maximum-likelihood approximation on purged alignments using the generalized time-reversible model in FastTree, v. 2.1.8 (10), with local support assessed by 1,000 resamples. Phylogenies were used for visualization of population structure, annotated with relevant metadata, and visualized using iTOL (11).

Plasmid marker screening

From the public domain, three IncX3-plasmids of identical size were identified, all with ≥3 point mutations compared with the IncX3-*bla*$_{NDM-5}$ outbreak plasmid. A 75-bp plasmid-specific oligonucleotide marker was designed to serve as a proxy for the IncX3-*bla*$_{NDM-5}$ plasmid in subsequent screening of short-read data:
pEhST1344_75bp:

AGAAGAATTTCCCCGCTTATTCGCACCTTCCCTAGGATGTCTTTTAATGAGTTCAGCAAATTTTCTGGCTGAAAA. Validation against sequences in the NCBI database identified matches exclusively to the same three IncX3 plasmids detected by full-length plasmid comparison, indicating high concordance between marker and outbreak plasmid sequence.

References

1. Bankevich A, Nurk S, Antipov D, Gurevich AA, Dvorkin M, Kulikov AS, *et al.* SPAdes: A new genome assembly algorithm and its applications to single-cell sequencing. J Comput Biol. 2012;19(5):455–77. http://dx.doi.org/10.1089/cmb.2012.0021 PMID:22506599

2. Larsen MV, Cosentino S, Rasmussen S, Friis C, Hasman H, Marvig RL, *et al.* Multilocus sequence typing of total-genome-sequenced bacteria. J Clin Microbiol. 2012 Apr;50(4):1355–61. http://dx.doi.org/10.1128/JCM.06094-11 PMID:22238442

3. Miyoshi-Akiyama T, Hayakawa K, Ohmagari N, Shimojima M, Kirikae T. Multilocus sequence typing (MLST) for characterization of *Enterobacter cloacae*. PLoS One. 2013 Jun 11;8(6):e66358. http://dx.doi.org/10.1371/journal.pone.0066358 PMID:23776664

4. Zankari E, Hasman H, Cosentino S, Vestergaard M, Rasmussen S, Lund O, *et al.* Identification of acquired antimicrobial resistance genes. J Antimicrob Chemother. 2012 Nov 1;67(11):2640–4. http://dx.doi.org/10.1093/jac/dks261 PMID:22782487

5. Carattoli A, Zankari E, García-Fernández A, Voldby Larsen M, Lund O, Villa L, *et al.* In Silico detection and typing of plasmids using PlasmidFinder and plasmid multilocus sequence typing. Antimicrob Agents Chemother. 2014 Jul;58(7):3895–903. http://dx.doi.org/10.1128/AAC.02412-14 PMID:24777092

6. Silva M, Machado MP, Silva DN, Rossi M, Moran-Gilad J, Santos S, *et al.* chewBBACA: A complete suite for gene-by-gene schema creation and strain identification. Microb Genomics. 2018;4(3):1-7. http://dx.doi.org/10.1099/mgen.0.000166 PMID:29543149

7. Germany: Ridom. *Enterobacter hormaechei* cgMLST. Available from: https://www.cgmlst.org/ncs/schema/Ehormaechei/

8. Prior K, Becker K, Brandt C, Cabal Rosel A, Dabernig-Heinz J, Kohler C, *et al*. Accurate and reproducible whole-genome genotyping for bacterial genomic surveillance with Nanopore sequencing data. J Clin Microbiol. 2025 Jul 9;63(7):e0036925. http://dx.doi.org/10.1128/jcm.00369-25 PMID:40511924

9. Sahl JW, Lemmer D, Travis J, Schupp JM, Gillece JD, Aziz M, *et al.* NASP: an accurate, rapid method for the identification of SNPs in WGS datasets that supports flexible input and output formats. Microb Genomics. 2016 Aug 25;2(8): e000074. http://dx.doi.org/10.1099/mgen.0.000074 PMID:28348869

10. Price MN, Dehal PS, Arkin AP. FastTree 2--approximately maximum-likelihood trees for large alignments. PLoS One. 2010 Mar 10;5(3):e9490. http://dx.doi.org/10.1371/journal.pone.0009490 PMID:20224823

11. Letunic I, Bork P. Interactive tree of life (iTOL) v3: an online tool for the display and annotation of phylogenetic and other trees. Nucleic Acids Res. 2016 Jul 8;44(W1):W242–5. http://dx.doi.org/10.1093/nar/gkw290 PMID:27095192
